# Mu opioid receptor activation in microglia enhances HIV-1 infection and HIV-infection-induced inflammatory responses

**DOI:** 10.3389/fimmu.2025.1628872

**Published:** 2025-10-06

**Authors:** Chelsey Skeete, Gabriel Sgambettera, Aldana D. Gojanovich, Xianbao He, Daniel Bryant, Mengwei Yang, Shreya Banerjee, Andrés A. Quiñones-Molina, Hisashi Akiyama, Gustavo Mostoslavsky, Andrew J. Henderson, Suryaram Gummuluru

**Affiliations:** ^1^ Department of Virology, Immunology, and Microbiology, Boston University Chobanian and Avedisian School of Medicine, Boston, MA, United States; ^2^ Boston University Chobanian and Avedisian School of Medicine, Center for Regenerative Medicine, Boston Medical Center, Boston, MA, United States; ^3^ Section of Infectious Diseases, Boston Medical Center, Boston, MA, United States

**Keywords:** HIV-1, opioids, inflammation, microglia, µ-opioid receptor

## Abstract

People living with HIV-1 (PWH) and chronically using opioids have elevated risks of developing HIV-associated neurological disorders (HAND) that are often correlated with persistent inflammation. Microglia, innate immune cells in the brain, are the principal HIV-1 reservoir in the central nervous system and regulate neuroinflammation. Our group previously showed that HIV-1 infection of induced pluripotent stem cell (iPSC)-derived microglia and viral intron-containing RNA (icRNA) expression triggers inflammatory responses. Microglia express μ opioid receptor, MOR, yet the immunomodulatory effects of opioids on HIV-1 infection in microglia are unclear. Here, we report that MOR activation impacts HIV-1 infection establishment and HIV-1-induced innate responses in microglia. Morphine pretreatment enhanced reverse transcription (RT), integration, viral transcription, and p24^Gag^ secretion in HIV-1-infected iPSC-derived microglia, which was blocked by treatment with naloxone, a MOR antagonist. In contrast, morphine treatment did not impact HIV-1 infection in MOR-deficient monocyte-derived macrophages, although, induced exogenous expression of MOR in macrophages conferred morphine-mediated enhancement of HIV-1 infection. Interestingly, viral transcriptome analysis by digital-drop PCR revealed selective enhancement of HIV-1 icRNA expression in morphine-exposed iPSC-derived microglia, which correlated with enhanced HIV-1 icRNA-induced secretion of IP-10 in MOR+ cells. Further, PI3K inhibitor, wortmannin, blocked morphine-mediated enhancement of HIV-1 replication and HIV-1 icRNA-induced IP-10 secretion, suggesting that MOR signaling and HIV-1 icRNA expression synergistically activate the PI3K-Akt signaling pathway in microglia to exacerbate virus-induced inflammatory responses.

## Introduction

HIV-1 remains a public health threat across the globe, with over 39.9 million people living with HIV-1 in 2023 (1). While new infections have declined by 39% as of 2023, primarily due to the expansion of antiretroviral therapy (ART) use, 1.3 million people were still newly diagnosed with HIV in 2023. Injection drug use is highly associated with HIV-1 infection, with intravenous drug use being a major route for HIV-1 infection (2). Globally, there are 11 million people who inject drugs (PWID), and 1 in 8 of these people are living with HIV-1 (3). The most common drug to be injected during non-medical usage is heroin, which is derived from morphine (4, 5). Heroin usage significantly influences opioid-dependency behaviors and raises the risk of developing opioid use disorders (OUD) (6), thus contributing to the establishment of the OUD and HIV-1 syndemic (7). In addition to intravenous routes, opioids such as morphine or fentanyl are also ingested via the oral route in pill form at higher rates in PWH than people without HIV (8). HIV-associated neurocognitive disorders (HAND) are a spectrum of neurocognitive impairments that are estimated to impact 30-50% of people with HIV-1 (PWH) globally (9). PWH who use injection drugs, such as opioids, are at higher risk for various co-morbidities, including neurocognitive disorders (10–12). Further, chronic opioid use has been shown to worsen neurocognition in PWH (13, 14). Therefore, understanding the underlying mechanisms by which opioids can exacerbate HAND is essential to the development of HAND treatments (15).

Microglia are the principal long-term cellular reservoir of HIV-1 in the CNS (16, 17). Despite ART, persistently infected microglia have been observed in PWH (18), and their longevity as brain resident macrophages might contribute to viral persistence (19). Microglia are regulators of neuroinflammation and secrete interferons and inflammatory cytokines such as interferon gamma-induced protein 10 (IP-10) and IL-1β in response to HIV-1 infection (15, 20–22). Secretion of inflammatory cytokines and interferons by microglia is suspected to damage surrounding neurons, contributing to the risk of HAND development (23–25). Specifically, type I interferons have been suggested to contribute to cognitive impairment (26), and IP-10 is associated with poor prognosis in PWH with HAND (27). Persistent neuroinflammation is highly associated with HAND (28–30). Further, opioids have been shown to worsen neuroinflammation by promoting microglial activation, lymphocyte infiltration by chemokine signaling, and blood-brain barrier (BBB) disruption in PWID (31). Concurrently, PWH and using injection opioids have higher levels of systemic inflammation than those who do not use injection drugs (32–34). However, the effects of opioid signaling on HIV infection and virus-induced inflammatory responses in the CNS are not well understood.

Human microglia express the three most common classes of opioid receptors, Mu (MOR), Delta (DOR), and Kappa (KOR) (35), as opposed to non-CNS resident myeloid cells such as macrophages, which have minimal opioid receptor expression (36–38). Opioid receptors are class A G-protein coupled receptors (GPCRs) activated by alkaloid opiates such as morphine (39, 40) that play a role in both reward and analgesic effects. Agonist binding results in receptor phosphorylation and activation that aids the recruitment of other signal transduction cascades, including mitogen-activated protein kinases (MAPKs), protein kinase C, and PI3-kinase (PI3K) dependent pathways (41–45). MOR expression is widespread in the CNS on both neurons and glial cells in the nucleus accumbens, hippocampus, amygdala, and spinal cord, though MOR activation in these cell types can contribute to divergent outcomes (46). For instance, opioid receptor activation in GABAergic neurons inhibits GABA release, which promotes dopamine release from dopaminergic neurons in CNS regions associated with reward, such as the ventral tegmental area (47–49). Opioid receptor activation in astrocytes is suggested to have anti-proliferative effects (50, 51), as well as inducing glutamate release (52). In contrast, opioid receptor activation in microglia, specifically by MOR agonists morphine and D-Ala (2)-mephe(4)-gly-ol(5))enkephalin (DAMGO), has been shown to enhance inflammatory effects (53, 54). MOR displays the highest binding affinity for morphine compared to DOR or KOR (55), and interestingly, ligation of MOR by morphine fails to promote receptor endocytosis and tolerance (56), which might contribute to the induction of prolonged signaling cascades. To date, the effects of morphine-induced MOR signaling on HIV-1 infection kinetics have not been defined in primary human microglia. Previous attempts to unravel mechanisms of HIV-1 interactions with microglia have either utilized murine microglia (57) or human cell lines with an unclear non-human origin that require transformation for maintenance in culture, which resulted in substantial loss in HIV-1 infectivity (58).

Here we investigated the role of morphine on HIV-1 infection in human microglia by using human induced pluripotent stem cell (iPSC) derived microglia. Recent studies by us and others have described that iPSC-derived microglia are permissive to HIV-1 infection, and the successful establishment of HIV-1 infection and expression of HIV-1 intron-containing RNA (HIV-1 icRNA) in iPSC-derived microglia induces inflammatory responses (59–63). In this report, we demonstrate that morphine enhances HIV-1 infection and viral icRNA expression in MOR+ iPSC-derived microglia, which is blocked by the opioid receptor antagonist, naloxone. In contrast, morphine does not significantly impact HIV-1 infection in monocyte-derived macrophages (MDMs), which have undetectable MOR expression. Significantly, overexpression of MOR in a human macrophage cell model, THP-1/PMA macrophages, rescues morphine-dependent enhancement of HIV-1 infection and subsequent HIV-1-infection-induced IP-10 secretion, suggesting that MOR expression is required for morphine-dependent modulation of HIV-1 infection in macrophages and microglia. Further PI3K inhibition suppresses morphine-mediated enhancement of HIV-1 infection and icRNA-induced inflammatory responses, suggesting that morphine-induced activation of MOR signaling synergizes with HIV-1 at the PI3K-Akt pathway to enhance infection kinetics and viral infection-induced inflammatory responses.

## Methods

### Cells

HEK293T cells (ATCC) and TZM-bl cells (NIH AIDS Reagent Program, Division of AIDS, NIAID, NIH: HRP-8129, contributed by Dr. John C. Kappes, Dr. Xiaoyun Wu, and Tranzyme Inc.) (Derdeyn, Decker et al., 2000) were maintained in culture with DMEM/10% FBS and 1% pen/strep (complete D10 media). THP-1 monocytic cells (ATCC, catalog #TIB-202) were cultured in RPMI1640/10% FBS/1% pen/strep (complete R10 media). To generate THP-1 macrophages, THP-1 monocytes were differentiated with PMA (100 nM, SIGMA, catalog # P8139) for 24h, washed, and returned to culture. PBMCs were isolated from fresh leukopaks obtained from NYBiologics from the fluffy top layer of Ficoll Paque Plus (Fisher, catalog# 45-001-750). CD14+ monocytes were isolated from PBMCs using CD14 bead isolation (Miltenyi, catalog# 130-045-101).

### Generation of MDMs

Human monocyte-derived macrophages (MDMs) were differentiated from CD14+ peripheral blood monocytes by culturing in RPMI 1640 containing 10% heat-inactivated human AB serum (Sigma, catalog # H4522) and recombinant human M-CSF (Peprotech, catalog # 300-25) (20 ng/mL) for 5 days before culturing in complete R10 media.

### Generation of iPSC-derived microglia

To generate iPSC-derived microglia, iPSCs are first differentiated toward a mesodermal, hematopoietic lineage using a protocol that consists of plating iPSCs at low confluency on Matrigel (Corning, catalog #354234)-coated plates in mTeSR (StemCell Technologies, catalog# 100-0276) for 2 days. Hematopoietic differentiation was initiated by replacing mTeSR with StemPro34 (Thermo Fisher Scientific, catalog# 10639011) media supplemented with Glutamax (Thermo Fisher Scientific, catalog#35050061), 450 nM alpha monothioglycerol (α-MTG) (Millipore Sigma, catalog# M6145-25ML), 88 ug/ml ascorbic acid (Sigma Aldrich, catalog #A4403-100MG), 200 g/ml transferrin (Millipore Sigma, catalog #10652202001), 0.1 mg/ml primocin (Fisher Scientific, catalog# ANT-PM-2), 5 ng/ml bone morphogenetic protein-4 (BMP4) (Peprotech, catalog #120-05ET-100UG), 50 ng/ml vascular endothelial growth factor (VEGF) (Peprotech, catalog # 100-20A-100UG) and 2µM CHIR99021 (Fisher Scientific, catalog #44-231-0), and transferring cells to hypoxic culture conditions (5% O_2_). On days 2 and 4, the media was changed, with 2µM CHIR99021 being switched out for 20 ng/ml basic fibroblast growth factor (bFGF) (StemCell Technologies, catalog #78003). On days 6 through 12, media was replaced every other day with StemPro34, containing Glutamax, 450 nM α-MTG, 50 μg/ml ascorbic acid, 150 g/ml transferrin, 0.1 mg/ml primocin, 10 ng/ml BMP4, 5 ng/ml VEGF, 5 ng/ml bFGF, 100 ng/ml SCF, 10 ng/ml FMS like tyrosine kinase 3 ligand (FLT3L) (R&D Systems, catalog #308-FKHB-050), 30 ng/ml thyroid peroxidase (TPO) (Peprotech, catalog #300-18-100UG), 30 ng/ml IL-3 (R&D Systems, catalog #203-IL-050/CF), 10ng/ml IL-6 (Peprotech, catalog #200-06), 5 ng/ml IL-11 (Peprotech, catalog #200-11), 25 ng/ml insulin like growth factor-1 (IFG1) (R&D Systems, catalog #291-G1-200) and 20 ng/ml sonic hedgehog recombinant protein (SHH) (Peprotech, catalog #100-45). Cells were switched back to normoxia conditions at day 8. At the end of the 12-day protocol, hematopoietic cells contain 25 - 65% (average 43%) CD34+CD45+ progenitor cells (64). Subsequently, the hematopoietic cells were harvested and differentiated toward microglia lineage using STEMdiff™ Microglia Differentiation Kit (StemCell Technologies, catalog #100-0019) and STEMdiff™ Microglia Maturation Kit (StemCell Technologies, catalog #100-0020). This protocol consists of 24 days of microglia differentiation followed by 4–10 days of microglia maturation, resulting in a highly pure population of microglia (> 90% CD45/CD11b-positive, 90% TREM2-positive microglia).

### Viruses

HIV-1 proviral plasmids, Lai/YU2-env (replication-competent, CCR5-tropic), and LaiΔenv/GFP (single-round GFP-expressing HIV-1 reporter virus) have been described previously (65, 66). Viruses were derived via calcium phosphate-mediated transient transfection of HEK293T cells with proviral plasmids, packaging plasmid (psPAX2), and VSV-G-expression plasmid (H-CMVG). As previously described, SIVmac Vpx containing VLPs were generated from HEK293T cells by co-transfection with SIV3+ packaging plasmid and H-CMVG (Goujon, Jarrosson-Wuillème et al., 2006). The plasmid pcDNA3.1/OPRM1, which expresses a flag-epitope-tagged MOR, was a gracious gift of the Ferré lab (67). To generate a flag-MOR-expressing retroviral expression plasmid, flag-OPRM1 orf was cloned into pLNCX using Hind III and Apa I restriction enzymes. LNC-MOR or empty LNCX retroviral particles were generated by calcium phosphate-mediated co-transfection of HEK293T cells with LNCX or LNC-MOR retroviral plasmids with packaging plasmid (pCL10A1) and envelope-expression plasmid (H-CMVG). Virus particles were harvested from HEK293T supernatants at two days post-transfection, filtered through a 0.45 µm filter, and concentrated via ultracentrifugation at 24,000 rpm for 1.5 hours at 4 °C over 20% sucrose using a SW32Ti rotor (Beckman Coulter). Virus pellets were resuspended in PBS and stored at -80 °C. To generate CD4+ T cell-derived Lai/YU-2env, CD4+ T cells were infected with Lai/YU2-env at MOI 0.1 by spinoculation at 2300 rpm for 1 hour, washed with PBS, and cultured in R10 media supplemented with 50U/mL IL-2. CD4+ T cell supernatants were harvested at days 3, 6, and 9 post-infection using the same ultracentrifugation steps described above. Infectious virus titers were measured on TZM-bI cells by measuring β-galactosidase activity as described previously in (68).

### Generation of MOR-expressing THP-1s

THP-1 monocytes were transduced with flag-MOR-expressing retrovirus particles and cultured in geneticin (Gibco, 10-131-027) (1 mg/mL)-containing complete R10 media. Surface expression of MOR was confirmed by flow cytometry and immunofluorescence analysis using anti-MOR polyclonal antibody (Novus Biologicals, catalog # 31180).

### Opioid treatment

Cells were treated with varying concentrations of morphine (NDC 00216-1307-08) or DMSO (vehicle control) for 24h before infection initiation. In some experimental conditions, cells were treated with 1µM naloxone (Sigma-Aldrich, catalog #BP548) for 1 hour before morphine was added.

### Infection

iPSC-derived microglia and MDMs were infected with CD4+ T cell-passaged Lai/YU-2env, and THP-1/PMA macrophages were infected with LaiΔenv/GFP/G. To increase the permissiveness of THP-1/PMA macrophages and MDMs to HIV-1 infection, infection conditions included either SIVmac Vpx-VLPs (5 ng of p27) or deoxyribonucleosides (dNs, 2.5mM, Sigma-Aldrich, catalog# D8668, D0776, T1895, D0901) for THP1/macrophages and MDMs, respectively. In some experiments, cells were pretreated with efavirenz (EFV, 1 µM, NIH AIDS Reagent Program) or raltegravir (Ral, 30 µM, Selleck Chemicals, catalog #50-615-1). DMSO (Sigma Aldrich, catalog #BP231-1) was used as a vehicle control. Cells were spinoculated with virus-containing supernatants for 1 hr at 2300 rpm at room temperature with various multiplicities of infection (MOI) ranging from 0.2 to 1. Cells were washed 2 hours after spinoculation to remove unbound virus. Cells were harvested 3 days post-infection, and cells and cell-free supernatants were processed for measurements of infection establishment (flow cytometry for GFP expression or p24^gag^ ELISA).

### RNA analysis

Total mRNA (100 ng), isolated from 5x10^5^ cells using a RNeasy Plus kit (Qiagen, catalog #74136), was reverse transcribed (Superscript IV, Invitrogen, catalog #18-090-010) using oligo(dT)20, and random hexamer primers. Target mRNA was quantified using Maxima SYBR Green (Thermo Scientific, catalog #FERK0242) for the following targets: OPRM1, HIV-1 msRNA, HIV-1 gRNA. The threshold cycle (Cq) was normalized to that of GAPDH using the ΔΔCt method (Rao, Huang et al., 2013). Primers for RT-PCR are listed in **Table 1**. For RT-ddPCR, FAM-conjugated probes were designed to distinguish HIV transcripts. RT-ddPCR probe design was based on those previously reported (69) and multiplexed to measure the frequency of probe sites simultaneously in ddPCR assays. Cycling conditions for ddPCR were described previously (70). Signals were identified using a Bio-Rad QX200 Droplet Digital PCR System and Quantasoft Data Analysis Software. The lists of primers and probes for RT-ddPCR are listed in **Tables 2** and **3**, respectively.

**Table 1:** List of primers for RT-PCR.

Gene name	Forward (5’…3’)	Reverse (5’…3’)
GAPDH	CAAGATCATCAGCA ATGCCT	AGGGATGATGTTCT GGAGAG
IP-10	AAAGCAGTTAGCAA GGAAAG	TCATTGGTCACCTT TTAGTG
OPRM1	CAGATACACCAAGAT GAAGAC	CCCATTAGGTAATTC ACACTC
Early RT	GGCTAACTAGGGAAC CCACTG	CTGCTAGAGATTTTCCAC ACTGAC
Late RT	TGTGTGCCCGTCTG TTGTGT	GAGTCCTGCGTCGA GAGAGC
HIV-1 gRNA	TGTGTGCCCGTCTG TTGTGT	CTCTCCTTCTAGCC TCCGCT
HIV-1 msRNA	GCGACGAAGACCT CCTCAA	GAGGTGGGTTGCTTTGA TAGAGA

**Table 2:** List of primers for RT-ddPCR.

Gene name	Forward	Reverse
LTR	GCCTCAATAAAGCT TGCCTTGA	GGGCGCCACTGCTAGAGA
Nef	GGTGGGAGCAGYATC TCGAGA	TGTAAGTCATTGGTCTT AAAGGTACCTGAGG
Env	TVTTCMTTGGGTTCT TRGGAGCAGCAGG	GCACTATRCCAGACAA TAVYTGTCTGGCCTGTACC
Gag	GACTAGCGGAGGCT AGAAGGAGAGA	CTAATTTTCCSCCDCTTA ATAYTGACG

**Table 3:** List of probes for RT-ddPCR.

Gene name	Probe	FAM or HEX
LTR	CCAGAGTCACACAACAGACGGGCACA	FAM
Nef	CCAGGCACAAKCAGCATT	FAM
Env	AGCACKATGGG	HEX
Gag	ATGGGTGCGAGA	HEX

### DNA analysis

Cells were lysed with DNA lysis buffer containing 10 mM EDTA (Bioworld, catalog #40120337, 100 mM NaCl (Fisher Scientific, catalog #S271-3), 0.5% (W/V) SDS (Boston Bioproducts, catalog #BM-230), and 10 mM Tris-Cl (pH 8.0) (Boston Bioproducts, catalog #BBT-75). Lysates were treated with proteinase K (New England Biolabs, catalog #P8107) for 15min at 56 °C. DNA was isolated from proteinase K-treated cell lysates using phenol: chloroform: isoamyl alcohol (Fisher Scientific, 15593031) extraction, precipitated with sodium acetate and ethanol, and resuspended in water. Total cell DNA was used for quantification of early and late RT products by qPCR (primers are listed in **Table 1**).

### Intact proviral DNA assay

Primers and probes targeting the 5’ (psi sequence) and 3’ regions (RRE/env) of HIV-1 provirus, conjugated to FAM and VIC fluorophores, respectively, were used as previously described (70, 71). The lists for IPDA primers and probes are listed in **Tables 4** and **5**, respectively. The dark probe recognized hypermutated Env sequences to define hypermutated provirus sequences. Hypermutated signals were detected as single positive droplets with FAM signals. RPP30 primers and probes were used to determine the cell number and DNA shearing index. Infections in the presence of EFV or RAL were used as controls to confirm that IPDA signals were dependent on reverse transcription and derived from integrated genomes. Cell numbers are estimated from the RPP30 and DNA shearing quantification.

**Table 4:** List of primers for IPDA.

Gene name	Forward	Reverse
Psi	CAGGACTCGGCTTG CTGAAG	GCACCCATCTCTCTC CTTCTAGC
Env	AGTGGTGCAGAGAG AAAAAAGAGC	GTCTGGCCTGTACCGT CAGC
RPP30-1	GATTTGGACCTGC GAGCG	GCGGCTGTCTCCACAAGT
RPP30-2	GACACAATGTTTGG TACATG GTTAA	CCATCTCACCAATCATTC TCCTTCCTTC

**Table 5:** List of probes for IPDA.

Gene name	Probe	FAM or HEX
Psi	TTTTGGCGTACTCACCAGT	FAM
Env (Intact)	CCTTGGGTTCTTGGGA	VIC
Env (Hypermut)	CCTTAGGTTCTTAGGAGC	Dark Probe
RPP30-1	CTGACCTGAAGGCTCT	HEX
RPP30-2	CTTTGCTTTGTATGTTGGCAGAAA	FAM

### Western blot analysis

THP-1/PMA macrophages were pretreated with 1 µM naloxone for 20min before 1 µM morphine or DMSO treatment for 7min. Cells were washed with ice-cold PBS and lysed with RIPA buffer (Boston Bioproducts, catalog #116TX) supplemented with protease inhibitors (Sigma-Aldrich, catalog # 4693159001). Lysates were clarified by centrifugation for 20min at 13,000 rpm. Protein concentrations were determined using Bradford reagent (Fisher Scientific, catalog #PI23246). Cell lysates containing 30 µg of total protein were separated by SDS-PAGE and transferred to nitrocellulose membranes. Membranes were blocked with Li-Cor Blocking Buffer (Fisher Scientific, catalog #NC1660556) before being probed with rabbit anti-phospho-Akt (Cell Signaling, catalog # 4060, 1:1000) or rabbit anti-phospho-MOR (Cell Signaling, catalog #3451, 1:1000) and mouse anti-β-actin antibody (Thermo-Fisher, catalog #AM4302, 1:5000). Staining was visualized with secondary antibodies: goat anti-mouse-IgG-DyLight 680 (Pierce, catalog #35568) and goat anti-rabbit-IgG-DyLight 800 (Pierce, catalog #35518). Membranes were stripped with stripping buffer (Invitrogen, catalog #46430) for 30min at room temperature and probed again for rabbit anti-pan-Akt (Cell Signaling, catalog #4691, 1:1000) and mouse anti-actin. Membranes were scanned with Odyssey CLx scanner (Li-Cor).

### Imaging

For THP-1/PMA macrophages, MDMs, and iPSC-derived microglia, cells cultured on coverslips were stained with Alexa594-conjugated wheat germ agglutinin (Thermo-Fisher, catalog #32464, 1:250) for 20 minutes at 4 °C to label the plasma membrane. Cells were washed with PBS and stained with a rabbit anti-MOR antibody (Novus Biologics, catalog #NBP1-31180 1:500) followed by an Alexa Fluor 488-conjugated goat anti-rabbit IgG (Invitrogen, catalog #A11070, 1:200). Cells were fixed with 4% paraformaldehyde (Boston Bioproducts, catalog#BM-155) followed by DAPI (49,6-diamidino-2-phenylindole; Sigma-Aldrich, catalog #D9542) staining to visualize nuclei. Stained cells were imaged with a Nikon SP5 confocal microscope. Images were analyzed with ImageJ (NIH).

### ELISAs

IP-10 secretion in culture supernatants was measured with a BD human IP-10 ELISA set (BD, catalog #B550926). IL-8 secretion in culture supernatants was measured with a BD human IL-8 ELISA set (BD, catalog# B555244). MCP-1 secretion in culture supernatants was measured with a BD human MCP-1 ELISA set (BD, catalog #B555179). The p24^Gag^ content in cell supernatant secretion was measured by an ELISA, as previously described (Akiyama, Miller et al., 2018). SIVmac Vpx VLPs were tittered using a commercial p27 ELISA (XpressBio, catalog #SK845).

### Cell viability assays

Cell viability was quantified by lactate dehydrogenase (LDH) measurements in cell culture supernatants using a commercial cytotoxicity assay (Fisher Scientific, catalog #PR-G1780).

## Results

### Morphine enhances HIV-1 infection establishment in microglia in a MOR-dependent manner

**Figure 1:**
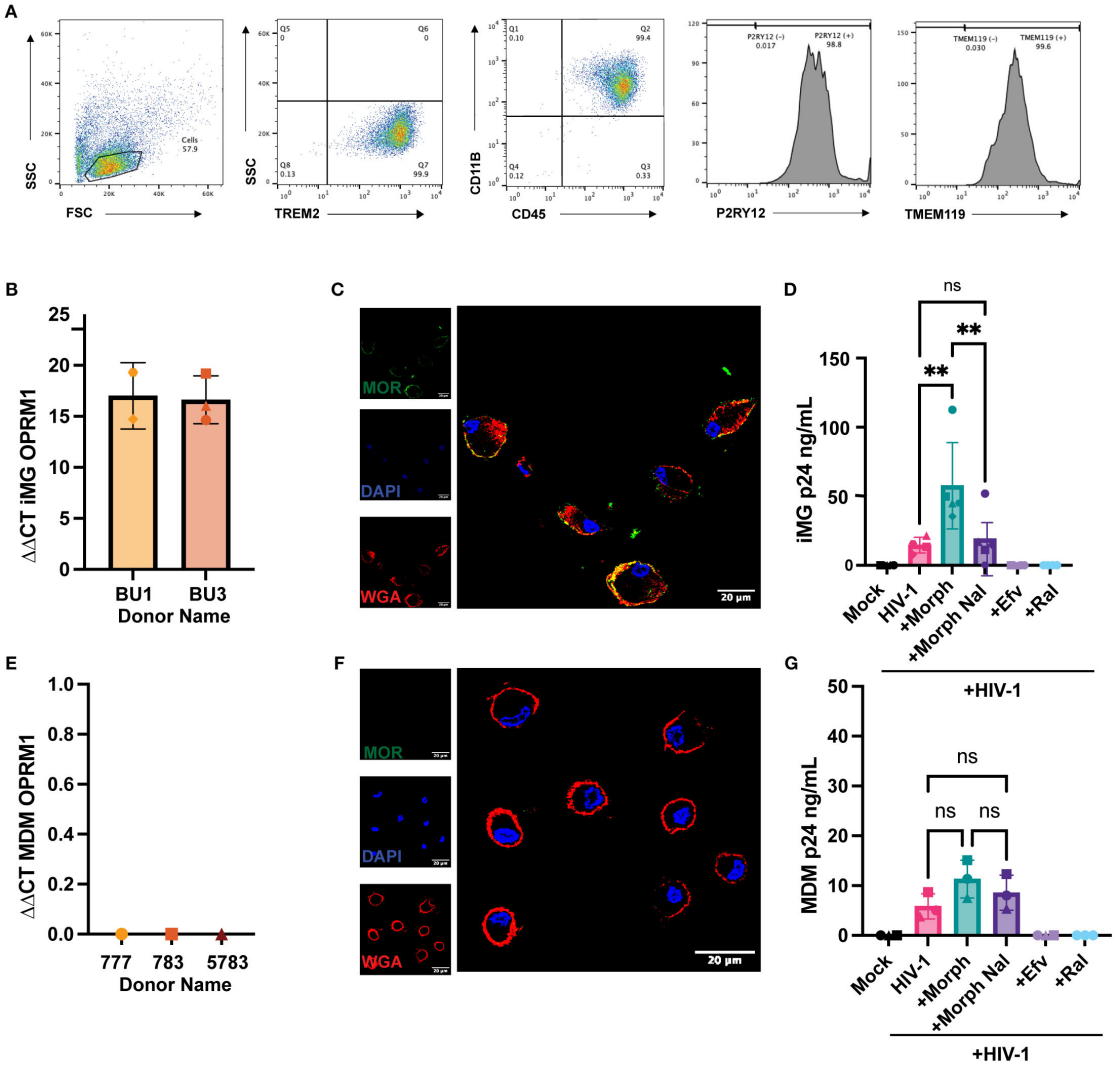
Morphine enhances HIV-1 infection in microglia. **(A)** Representative flow cytometry plots of TREM2, CD45, CD11B, P2RY12, and TMEM119 expression in iPSC-derived microglia. **(B)** OPRM1 mRNA expression was measured by RT-qPCR and normalized to GAPDH in iPSC-derived microglia **(C)** Representative immunofluorescence images of MOR surface expression: stained nucleus (DAPI, blue), plasma membrane (wheat germ agglutinin, red), and MOR (green) in iPSC-derived microglia. **(D)** iPSC-derived microglia were pretreated with DMSO (vehicle control) or naloxone (1µM) for 1 hr before treatment with morphine (1 µM) for 24hr. Cells were washed and infected with HIV-1(Lai/YU2-env, MOI 0.2). Supernatants were harvested 3 days post-infection for p24^Gag^ measurements by ELISA. **(E)** OPRM1 mRNA expression was measured by RT-PCR and normalized to GAPDH in MDMs. **(F)** Representative immunofluorescence images of MOR surface expression: stained nucleus (DAPI, blue), plasma membrane (wheat germ agglutinin, red), and MOR (green) in MDMs. **(G)** MDMs were pretreated with DMSO (vehicle control) or naloxone (1 µM) for 1 hr before 1 µM morphine was treated for 24 hr. Cells were washed and infected with HIV-1 (Lai/YU2-env, MOI 1, with SIVmac Vpx VLPs (5ng p27)). Supernatants were harvested 3 days post-infection for p24^Gag^ measurements by ELISA in MDMs. The means +/- SEM are shown, and each symbol represents an independent experiment for iPSC-derived microglia. P values: one-way ANOVA followed by the Tukey-Kramer posttest; **, p<0.01 **(A-G)**.

Microglia express opioid receptors, though the consequences of opioid exposure on HIV-1 infection establishment in microglia have not been mechanistically characterized, primarily due to limited access to primary human microglia. To overcome problems with cell availability, we chose to determine opioid effects on iPSC-derived microglia, which are phenotypically similar to CNS-resident microglia. (Pandya, Shen et al., 2017) Importantly, we have previously shown that iPSC-derived microglia are susceptible to HIV-1 infection *in vitro* (60). We used an iPSC-derived microglia differentiation protocol that utilized hematopoietic progenitors on two donor-derived cell lines, BU1 and BU3. Following cell differentiation protocol, > 99% of the CD45+ cells expressed microglial markers CD11b, TREM2, P2RY12, and TMEM119 (**Figure 1A**). Similar to CNS-resident microglia (Maduna, Audouard et al., 2019), iPSC-derived microglia expressed MOR mRNA (**Figure 1B**), and cell surface MOR expression was confirmed by confocal immunofluorescence microscopy (**Figure 1C**). MOR expression overlapped with plasma membrane staining and was also located at subcellular compartments proximal to the plasma membrane (**Figure 1C**). iPSC-derived microglia were treated with morphine prior to infection with CCR5-tropic replication-competent HIV-1 (Lai-YU2/env). Interestingly, morphine pretreatment enhanced HIV-1 replication in MOR+ iPSC-derived microglia. We observed ~3-fold enhancement in p24^Gag^ secretion at 3 days post-infection (**Figure 1D**). Morphine-mediated enhancement of p24^Gag^ secretion was suppressed upon naloxone pre-treatment, a MOR antagonist, suggesting that pro-viral effects of morphine required MOR activation in iPSC-derived microglia. The expression of opioid receptors on human macrophages has remained controversial. While some studies have documented MOR mRNA expression in peripheral blood mononuclear cells (PBMCs) and monocytes (Chuang, Killam et al., 1995), expression of MOR protein has not been observed in human macrophages. In agreement with some of the previously published studies (36, 37), monocyte-derived macrophages (MDMs) did not express MOR mRNA or protein (**Figures 1E, F**). In correlation with the lack of MOR expression, morphine pre-treatment did not significantly impact p24^Gag^ production from MOR-deficient MDMs (**Figure 1G**). Therefore, morphine increases HIV-1 virion release in microglia but not from macrophages, suggesting that morphine enhancement of HIV-1 infection is dependent on cell-surface MOR expression.

### Exogenous expression of MOR confers morphine-mediated enhancement of HIV-1 infection in macrophages

**Figure 2:**
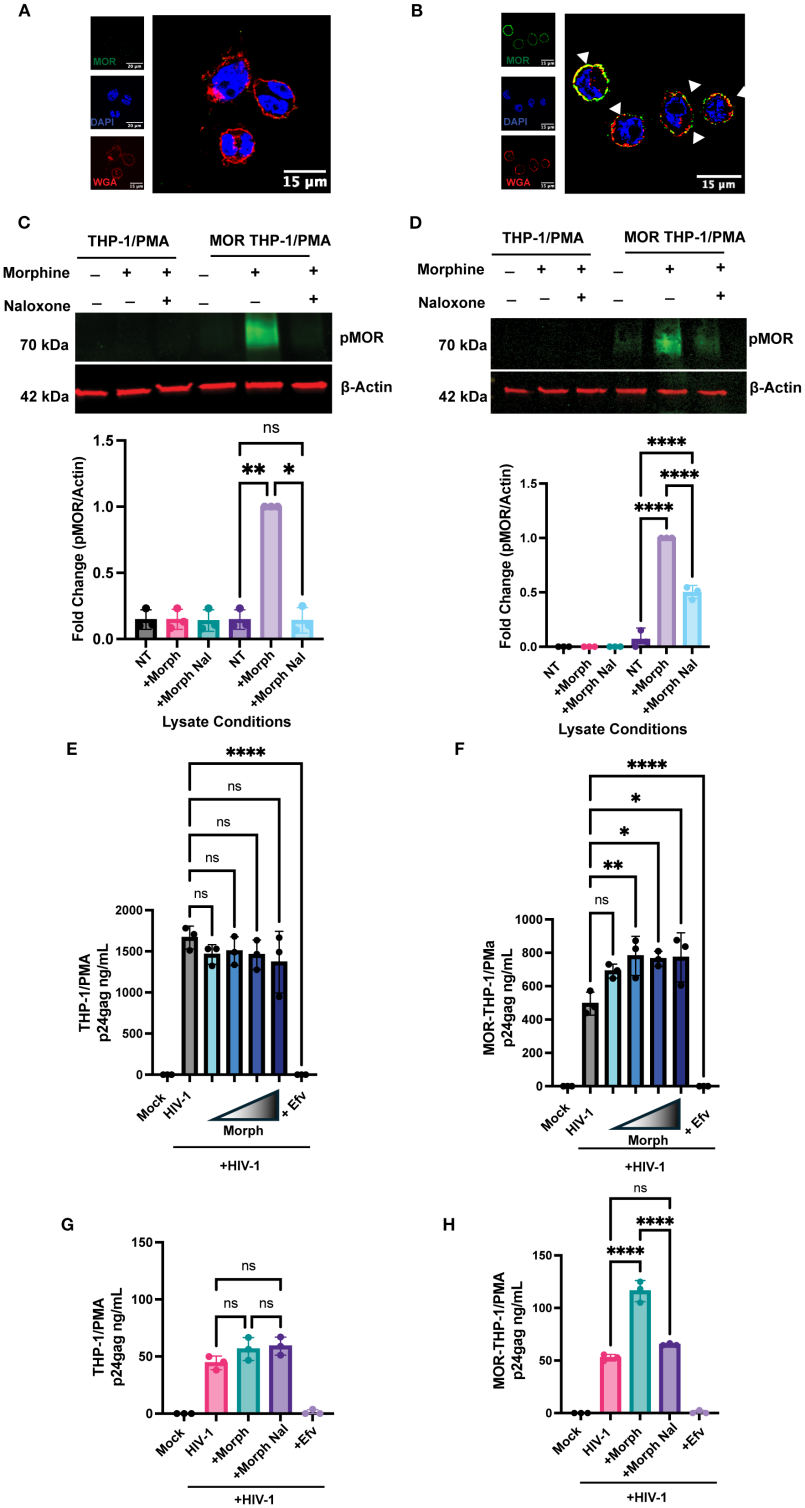
Exogenous MOR expression confers MOR-dependent enhancement of HIV-1 infection in macrophages. **(A, B)** Representative immunofluorescence images of MOR surface expression: stained nucleus (DAPI, blue), plasma membrane (wheat germ agglutinin, red), and flag-MOR (green) in **(A)** parental THP-1/PMA and **(B)** MOR-THP-1/PMA macrophages. Phospho-MOR (pMOR) expression in parental THP-1/PMA macrophages and MOR-THP-1/PMA macrophages with 7 min **(C)** and 24h **(D)** morphine treatment. pMOR band intensities were quantified and normalized to actin and to the no treatment (NT) group. THP1/PMA **(E)** or MOR-THP1/PMA **(F)** macrophages were pretreated with DMSO (vehicle control) or increasing concentrations (0.01, 0.1, 1, or 10 µM) of morphine for 24 hours. **(G, H)** Alternatively, cells were pretreated with naloxone (1 µM) prior to morphine (1 µM) treatment. Cells were washed and co-infected with HIV-1 (LaiΔEnvGFP/G, MOI 1, in **(E, F)** and MOI 0.3 in **(G, H)**) and SIVmac Vpx VLPs (5 ng p27). Supernatants were harvested 3 days post-infection for p24^Gag^ measurements by ELISA. The means +/- standard error of the SEM are shown, and each symbol represents an independent experiment. p values: one-way ANOVA followed by the Tukey-Kramer post-test; *, p< 0.05; **, p< 0.01; ****, p< 0.0001 **(A-H)**.

To confirm the requirement of MOR expression for morphine-dependent enhancement of HIV-1 infection, we retrovirally transduced THP-1 monocytes to constitutively express MOR and differentiated cells to macrophages by phorbol 12-myristate 13-acetate (PMA) treatment. While parental THP-1/PMA macrophages do not express MOR mRNA (**Supplementary Figure 1A**) or protein (**Figure 2A**), exogenous MOR expression by retroviral transduction in THP-1/PMA macrophages resulted in robust cell surface MOR expression (**Figure 2B**). We confirmed functional MOR expression in THP-1/PMA macrophages as morphine treatment induced phosphorylation of MOR, which was blocked by naloxone (**Figures 2C, D**). To determine the effects of morphine on HIV-1 infection, cells were pre-treated with increasing concentrations of morphine prior to virus exposure. While morphine did not affect p24^Gag^ production in parental THP-1/PMA macrophages (**Figure 2E**), morphine pre-treatment of MOR-THP-1/PMA macrophages significantly enhanced p24^Gag^ secretion in a dose-dependent manner, with the induction peaking between 0.1 and 1 µM morphine (**Figure 2F**). Hence, we used a dose of 1 µM morphine for all subsequent infection experiments. Morphine at 1 µM did not impact p24^Gag^ secretion in THP-1/PMA macrophages (**Figure 2G**). Importantly, morphine-mediated induction of p24^Gag^ secretion in MOR-THP-1/PMA macrophages was suppressed by pre-treatment with the MOR antagonist, naloxone (**Figure 2H**). These findings suggest that morphine enhances HIV-1 infection and virion release in a MOR-dependent manner.

### Morphine enhances HIV-1 reverse transcription in MOR-expressing cells

**Figure 3:**
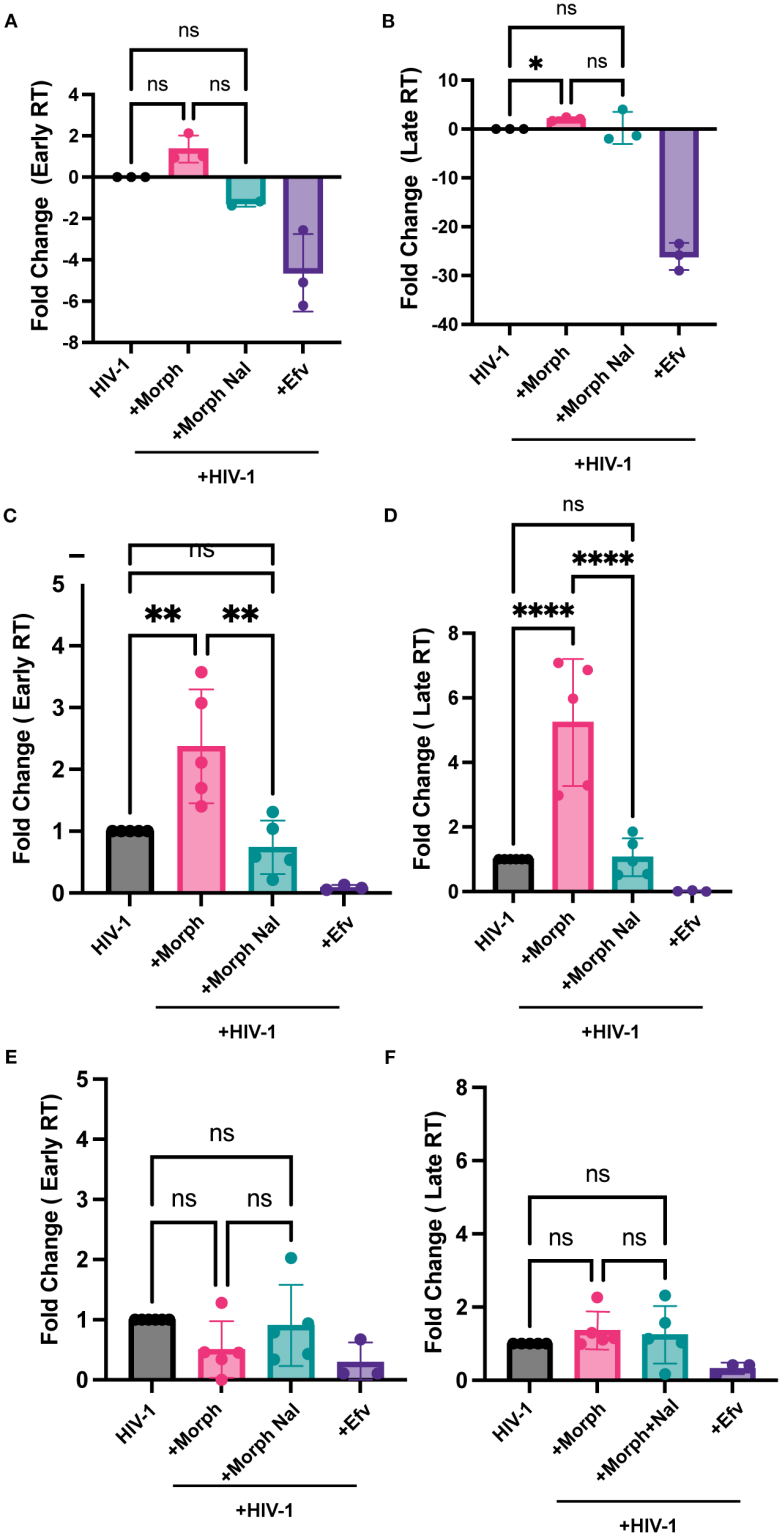
Morphine enhances HIV-1 reverse transcription in a MOR-dependent manner. iPSC-derived microglia **(A, B)**, MOR-THP-1/PMA **(C, D)**, or THP-1/PMA **(E, F)** macrophages were pretreated with DMSO (vehicle control) or naloxone (1µM) for 1 hr prior to morphine (1µM) treatment for 24 hr. Cells were washed and co-infected with HIV-1 (LaiΔEnvGFP/G, MOI0.3) and SIVmac Vpx VLPs (5 ng p27). THP/PMA macrophages were lysed at 8h and 24h post-infection for measurement of early **(C, E)** and late RT **(D, F)** products by qPCR. iPSC-derived microglia were lysed 3 days post-infection for measurement of early **(A)** and late RT **(B)**. The means +/- SEM are shown, and each symbol represents an independent experiment. p values: one-way ANOVA followed by the Tukey-Kramer posttest; *, p< 0.05; **, p< 0.01; ****, p< 0.0001 **(A-D)**.

Since MOR activation enhanced HIV-1 replication in MOR-expressing cells, we sought to systematically determine the specific step of the HIV-1 life cycle that was modulated by morphine. First, we assessed the effects of MOR activation on early steps of reverse transcription in iPSC-derived microglia and MOR-expressing or MOR-deficient (parental) THP1/PMA macrophages. Morphine pretreatment enhanced early and late RT in iPSC-derived microglia, though only increase in late RT was statistically significant (**Figures 3A, B**). Further, morphine treatment of MOR/THP-1/PMA macrophages significantly enhanced both early and late RT which was blocked by naloxone pretreatment (**Figures 3C, D**). In contrast, morphine pretreatment did not significantly impact early (**Figure 3E**) or late RT steps (**Figure 3F**) in parental THP-1/PMA macrophages. These findings suggest that MOR activation by morphine enhances HIV-1 reverse transcription, which can be blocked by a MOR antagonist.

### Morphine enhances intact proviral establishment in MOR-expressing cells

**Figure 4:**
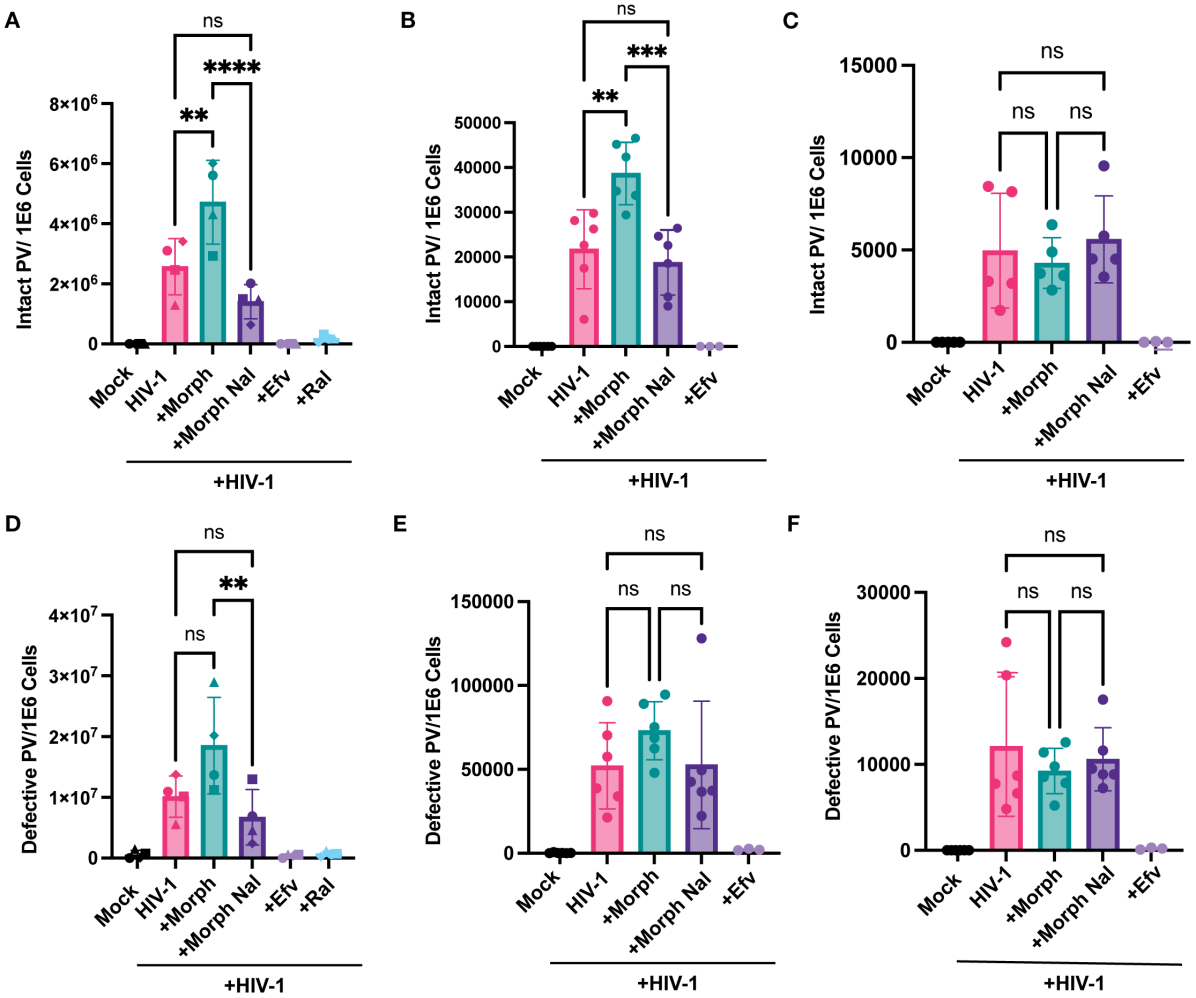
Morphine enhances intact but not defective provirus establishment in a MOR-dependent manner. iPSC-derived microglia and THP-1/PMA macrophages, pretreated with DMSO (vehicle control) or naloxone (1 μM) for 1 hr prior to morphine (1 µM) treatment for 24hr, were infected with HIV-1 (Lai-YU2-env, MOI0.2) for iPSC-derived microglia or LaiΔEnvGFP/G, MOI0.3, with SIVmac Vpx VLP (5 ng p27) for THP1/PMA macrophages. Cells were lysed for DNA extraction 3 days and 1 day post-infection for iPSC-derived microglia and THP-1/PMA macrophages, respectively, and used for measurement of intact and defective proviruses by IPDA. A parallel reaction to detect the host cell gene RPP30 was used as a correction for DNA shearing. Quantification of intact HIV-1 proviruses in **(A)** iPSC-derived microglia, **(B)** MOR-THP-1/PMA macrophages, and **(C)** THP-1/PMA macrophages. Quantification of defective HIV-1 proviruses in **(D)** iPSC-derived microglia, **(E)** MOR-THP-1/PMA, **(F)** THP-1/PMA macrophages. The means +/- SEM are shown, and each symbol represents an independent experiment for iPSC-derived microglia and THP-1/PMA. p values: one-way ANOVA followed by the Tukey-Kramer posttest; **, p< 0.01; ***, p< 0.001; ****, p< 0.0001 **(A–F)**.

Since MOR activation enhanced reverse transcription efficiency in MOR-expressing cells, we assessed the effects of MOR activation on the next step, namely, provirus establishment. We utilized Intact Proviral DNA Assay (IPDA) using digital droplet PCR (ddPCR) analysis (70, 72) to determine the number of proviruses in both MOR-expressing (iPSC-derived microglia and MOR- THP-1/PMA macrophages) and cells lacking MOR expression (parental THP-1/PMA macrophages) upon HIV-1 infection in the presence or absence of morphine and naloxone. Genomic DNA, harvested 3 days post-infection, served as a template for IPDA to quantify both intact and defective (lacking or mutated detection of env or Psi sequences) proviruses. Morphine pre-treatment significantly enhanced the numbers of intact proviruses (2-fold) in HIV-1-infected iPSC-derived microglia (**Figure 4A**) and MOR-THP1/PMA macrophages (**Figure 4B**), which was suppressed by treatment with naloxone. However, morphine treatment did not impact intact proviral establishment in parental THP-1/PMA macrophages (**Figure 4C**), suggesting that MOR expression and activation were required for morphine-induced HIV-1 reverse transcription and integration enhancements.

HIV-1 infection generates both intact and defective proviruses, with the defective proviruses encompassing by far the predominant fraction of the provirus load *in vitro* and *in vivo* (70, 72, 73). Interestingly, although statistically not significant, there was a trend towards enhanced defective proviral establishment in iPSC-derived microglia and MOR-THP-1/PMA upon morphine treatment, which was inhibited by naloxone (**Figures 4D, E**), while morphine had no impact on defective proviral establishment in THP-1/PMA macrophages (**Figure 4F**). In fact, the ratio of intact to defective proviruses was not affected by morphine treatment in iPSC-derived microglia and MOR THP-1/PMA macrophages (**Supplementary Figures S2 B, F, J**). These results indicate that morphine treatment did not significantly impact error rates of HIV-1 integration steps. Rather, these findings suggest that morphine exposure enhances the establishment of intact proviruses in MOR-expressing cells.

### Morphine enhances abundance of HIV-1 intron-containing transcripts in MOR-expressing cells

**Figure 5:**
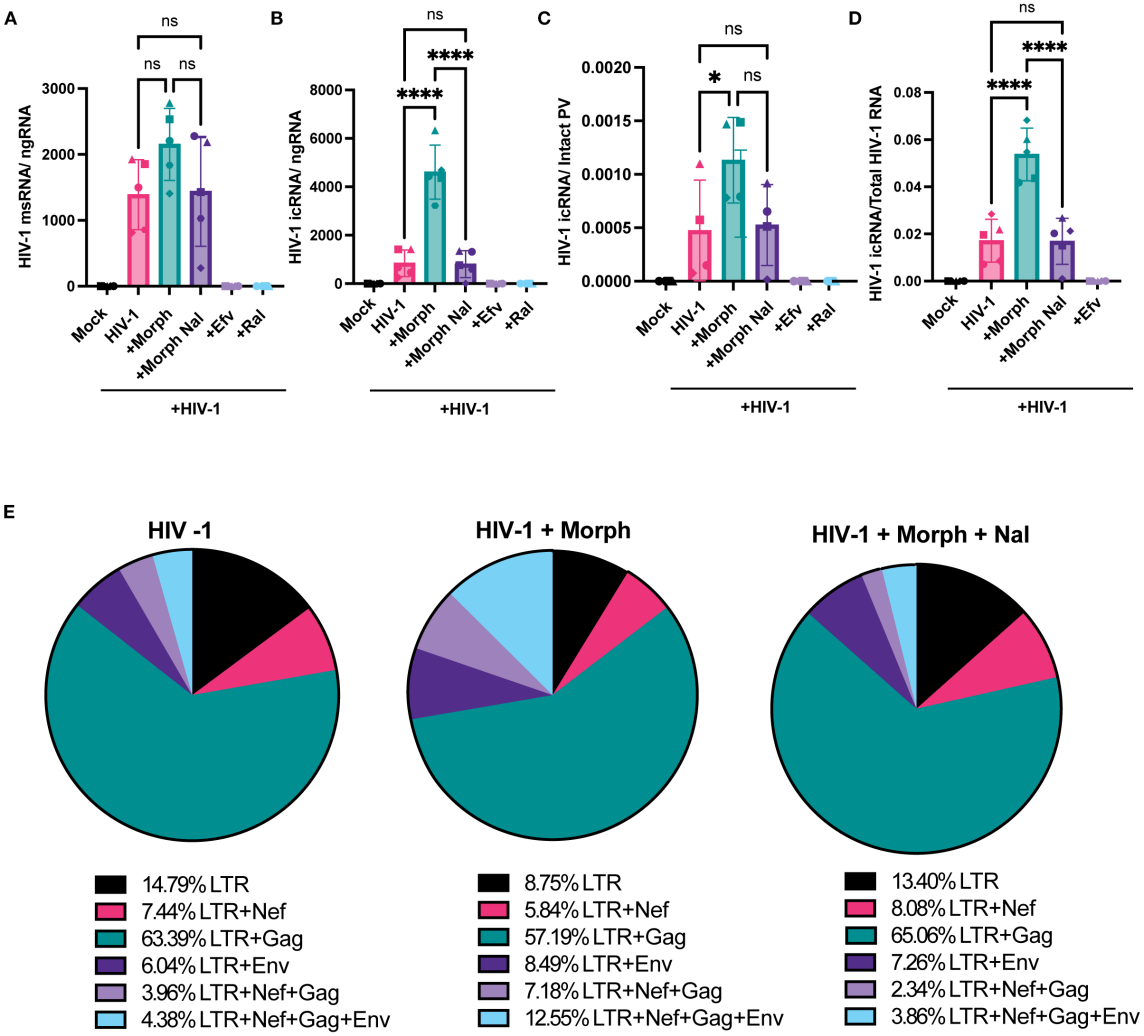
Morphine enhances HIV-1 full-length icRNA transcript abundance and alters transcriptomic diversity in microglia. iPSC-derived microglia were pretreated with DMSO (vehicle control) or naloxone (1 µM) for 1 hr prior to morphine (1 µM) treatment for 24 h, and then infected with HIV-1 (Lai-YU2-env, MOI 0.2). Cells were lysed for RNA extraction 3 days post-infection. **(A)** Quantification of msRNA (LTR+Nef), and **(B)** icRNA (LTR+Env+Nef+Gag, full-length transcripts) in iPSC-derived microglia were determined by RT-ddPCR and reported as viral RNA copies/ng of RNA input. **(C)** HIV-1 icRNA copy numbers were normalized to those of intact proviruses in iPSC-derived microglia. **(D)** Quantification of the ratio of full-length icRNA transcripts out of all transcripts detected in iPSC-derived microglia. **(E)** Pie charts displaying the mean percentages of LTR containing transcripts in iPSC-derived microglia infected in the absence (HIV-1) or presence of morphine (HIV-1+ Morph) or both morphine and naloxone (HIV-1+ Morph + Nal). The means +/- SEM are shown, and each symbol represents an independent experiment (iPSC-derived microglia and THP/PMA macrophages) or cells from an independent donor (MDMs). p values: one-way ANOVA followed by the Tukey-Kramer posttest; *, p< 0.05; ****, p< 0.0001 **(A–D)**.

Several studies have demonstrated the extensive diversity of the viral transcriptome in HIV-1 infected cells (74, 75), though only a subset of these viral transcripts encodes for viral proteins essential for the completion of the viral life cycle. Drugs-of-abuse, including opioids, can regulate HIV-1 gene expression via diverse mechanisms, including activation of transcription factors, NF-kB (76) and AP-1 (77), and alleviating RNA polymerase II pausing to promote polymerase activity (78), though the direct requirement for MOR activation and signaling has not been addressed. Hence, we next sought to determine the effects of acute morphine treatment on proviral transcription in MOR-expressing cells. We quantified multiply spliced, partially spliced, and full-length unspliced transcripts by using a previously described RT-ddPCR assay (79). RT-ddPCR simultaneously quantifies both spliced and unspliced LTR-containing transcripts by multiplexing probes for transcripts spanning the 5’ LTR, nef, env, or gag regions. While morphine treatment modestly enhanced levels of multiply spliced transcripts in iPSC-derived microglia (**Figure 5A**), there was a significant enhancement in unspliced RNA (which we term HIV-1 icRNA) expression by ~5.7 fold, which was blocked by naloxone treatment (**Figure 5B**). Furthermore, morphine-induced HIV-1 icRNA expression remained significantly elevated even after accounting for an increased number of proviruses in morphine-treated iPSC-derived microglia (**Figure 5C**), suggesting that acute morphine exposure in microglia selectively enhances HIV-1 icRNA expression. In addition to assessing the abundance of LTR-containing transcripts, analysis of viral transcript diversity in HIV-infected iPSC-derived microglia revealed that morphine selectively enhanced the ratio of full-length unspliced HIV-1 icRNA to that of the total viral transcriptome by ~3-fold (**Figure 5D**). In contrast to increases in longer transcripts such as full-length HIV-1 icRNA and env deficient 5’LTR+Nef+Gag transcript, morphine pretreatment decreased the abundance of shorter LTR-containing transcripts such as 5’LTR and 5’LTR+Gag in iPSC-derived microglia (**Figure 5E**). However, ratios of all LTR-containing transcripts remained similar across the no-treatment and morphine+naloxone conditions (**Figure 5E**). These results suggest that morphine enhances HIV-1 full-length unspliced icRNA abundance by not only modulating splicing efficiency but also alleviating pausing at promoter-proximal regions of viral LTR to favor transcriptional elongation and expression of full-length transcripts in microglia.

**Figure 6:**
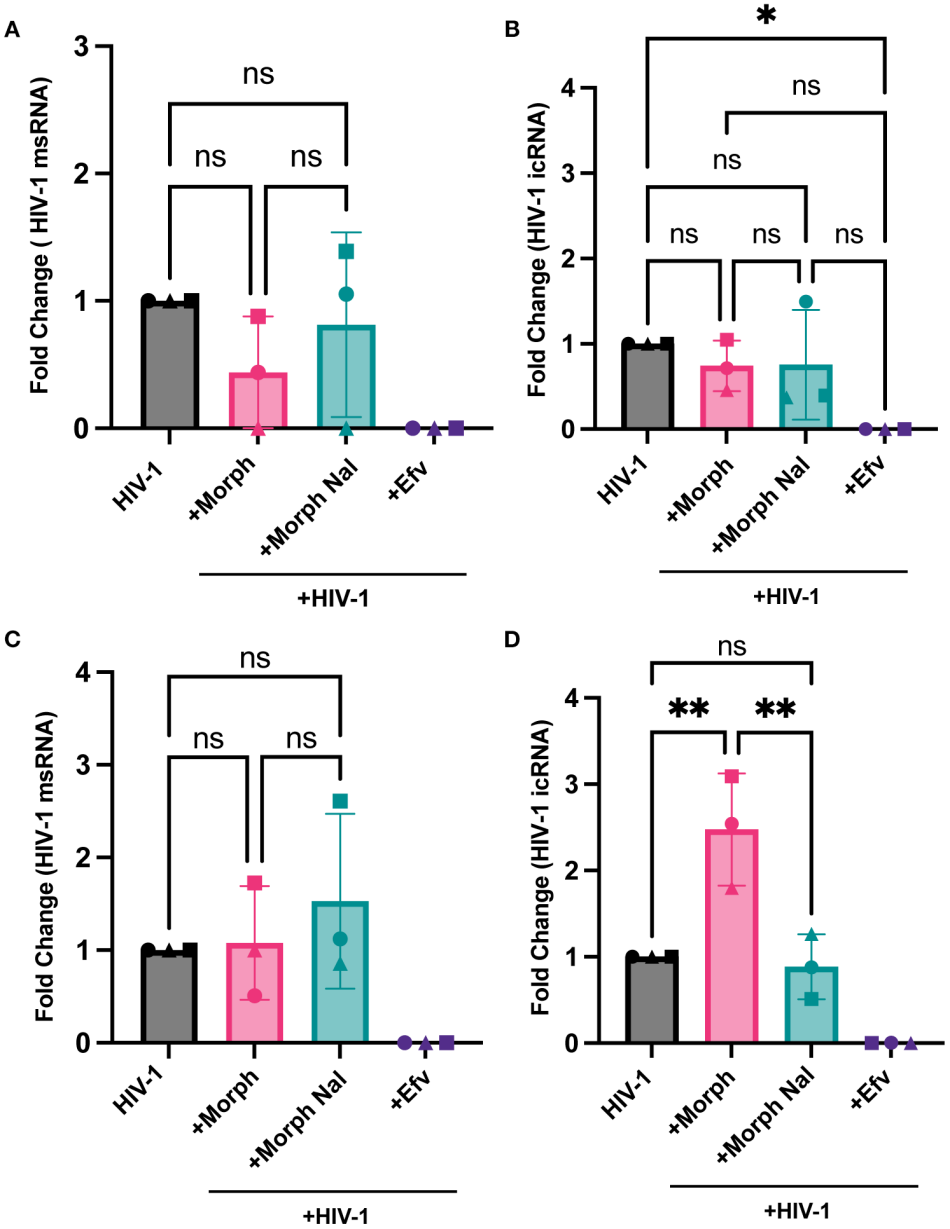
MOR overexpression promotes morphine-dependent enhancement of HIV-1 icRNA expression in macrophages. THP-1/PMA **(A, B)** or MOR-THP-1/PMA **(C, D)** macrophages were pretreated with DMSO (vehicle control) or naloxone (1µM) for 1 hr prior to morphine (1µM) treatment for 24hr. Cells were washed and infected with HIV-1 ((LaiΔEnvGFP/G, MOI0.3, with SIVmac Vpx VLP (5 ng p27)). Cells were lysed for RNA extraction 3 days post-infection. Levels of msRNA **(A, C)** and icRNA **(B, D)** in THP-1/PMA **(A, B)** or MOR-THP-1/PMA **(C, D)** macrophages were quantified by RT-qPCR. The means +/- SEM are shown, and each symbol represents an independent experiment. p values: one-way ANOVA followed by the Tukey-Kramer posttest; *, p< 0.05; **, p< 0.01 **(A-D)**.

We next assessed if morphine modulated HIV-1 transcription in macrophages in the presence or absence of MOR expression. While morphine-mediated enhancement of multiply-spliced (**Figure 6A**) and unspliced viral transcripts (**Figure 6B**) was not observed in parental THP-1/PMA (MOR-deficient) macrophages, morphine significantly enhanced full-length icRNA expression in MOR-THP-1/PMA macrophages (2.5 fold, **Figure 6D**). Surprisingly, msRNA expression was not impacted by morphine treatment in MOR-THP-1/PMA macrophages (**Figure 6C**). Importantly, morphine-induced enhancement of icRNA expression in MOR-THP-1/PMA macrophages was suppressed by naloxone (**Figure 6D**), suggesting that MOR expression and activation is responsible for the enhanced HIV icRNA expression.

### Morphine treatment enhances innate immune responses

**Figure 7:**
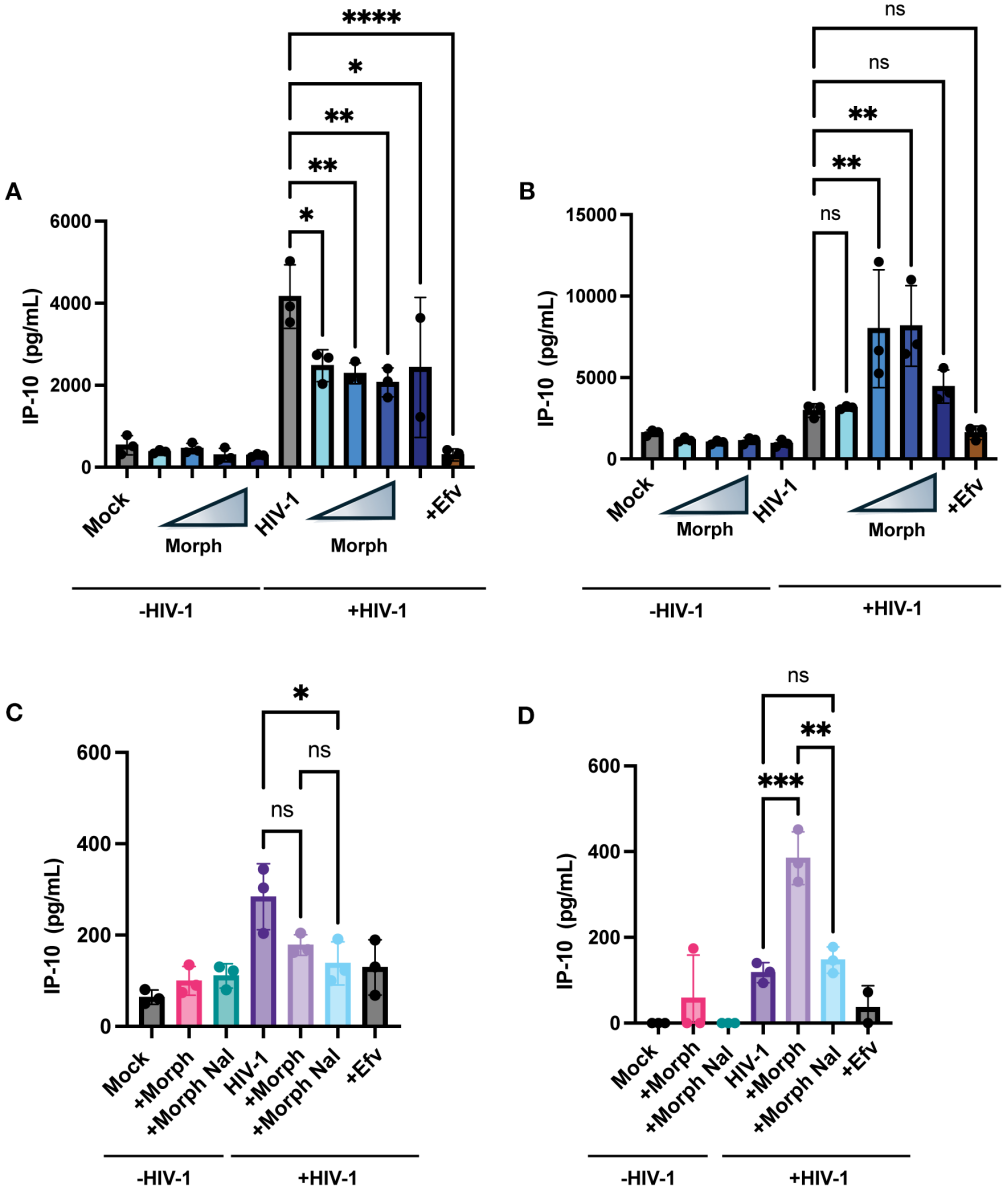
Exogenous MOR expression promotes morphine-dependent enhancement of HIV-1-induced IP-10 secretion in macrophages. THP-1/PMA **(A)** and MOR-THP-1/PMA **(B)** macrophages were pretreated with DMSO (vehicle control) or with increasing concentrations of morphine (0.01, 0.1, 1, and 10 µM) for 24hr. Cells were washed and co-infected with HIV-1 (LaiΔEnvGFP/G, MOI 1) and SIVmac Vpx VLPs (5 ng p27). IP-10 content in supernatants harvested 3 days post-infection was determined by an ELISA. Alternatively, cells were pretreated with naloxone (1 µM) prior to morphine (1 µM) treatment in THP-1/PMA **(C)** and MOR-THP-1/PMA macrophages **(D)**. The means +/- SEM are shown, and each symbol represents an independent experiment. p values: one-way ANOVA followed by the Tukey-Kramer posttest; *, p< 0.05; **, p< 0.01; ***, p< 0.001; ****, p< 0.0001 **(A–D)**.

Our previous studies have shown that *de novo* transcribed HIV-1 icRNA in both macrophages and microglia induce inflammatory responses, such as the interferon γ-inducible protein 10 (IP-10) (80). Hence, we sought to determine if morphine enhances IP-10 secretion in a MOR-dependent manner. THP-1/PMA macrophages were pretreated with increasing concentrations of morphine (0.01, 0.1, 1, or 10 µM) before infection with HIV-1, and IP-10 secretion was measured by ELISA on supernatants 3 days post-infection. While morphine modestly reduced IP-10 secretion in parental THP-1/PMA macrophages (**Figure 7A**), significant enhancement was observed in MOR-THP1/PMA macrophages in a dose-dependent manner, peaking at 1µM morphine (**Figure 7B**). Morphine treatment at 1µM modestly lowered IP-10 secretion in THP-1/PMA macrophages (**Figure 7C**). Moreover, the enhancement in IP-10 secretion (~3.4 fold) observed in morphine (1 µM) treated HIV-1 infected MOR-THP-1/PMA macrophages was blocked by naloxone (**Figure 7D**). These results suggest that MOR expression promotes morphine-dependent enhancement of IP-10 secretion during HIV-1 infection.

### HIV-1 infection and opioid signaling synergize at the PI3K/Akt pathway

**Figure 8:**
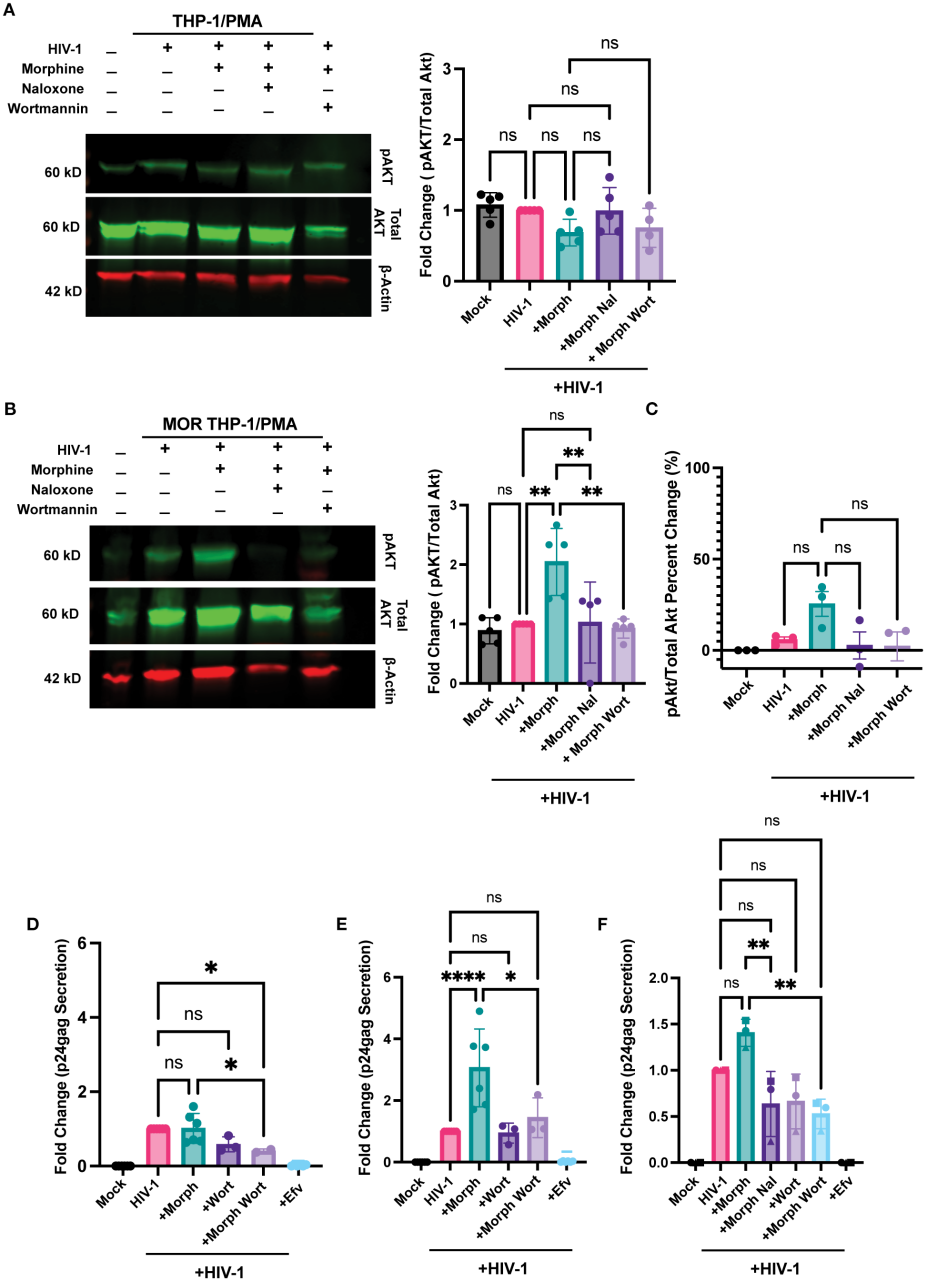
PI3K inhibition abrogates morphine-mediated enhancement of HIV-1 infection. **(A, B)** Western blot analysis of phosphorylated Akt (pAkt), total Akt, and β-actin on lysates from THP-1/PMA **(A)** or MOR-THP-1/PMA **(B)** macrophages. pAkt quantification on lysates from iPSC derived microglia by ELISA **(C)**. THP-1/PMA macrophages **(D)**, MOR-THP-1/PMA macrophages **(E)** and iPSC-derived microglia **(F)** were pretreated with combinations of morphine (1 µM) naloxone (1 µM) or wortmannin (0.1 µM) before infection with HIV-1. THP-1/PMA macrophages were co-infected with LaiΔEnvGFP/G (MOI 1) and SIVmac Vpx VLPs (5 ng p27), while iPSC-derived microglia were infected with Lai/YU2-env (MOI 0.2). The p24^Gag^ content in cell supernatants, harvested 3 days post-infection, was determined by an ELISA. The means +/- SEM are shown, and each symbol represents an independent experiment. p values: one-way ANOVA followed by the Tukey-Kramer posttest; *, p< 0.05; **, p< 0.01; ****, p< 0.0001 **(A–E)**.

Next, we sought to identify the pathway by which opioid signaling and HIV-1 infection synergized to enhance infection and inflammatory responses. MOR signaling has previously been shown to activate the PI3K/Akt pathway (81, 82). Additionally, HIV-1 infection has been shown to engage the PI3K/Akt pathway in macrophages (83) and T cells (84). Therefore, we sought to determine whether ligation of MOR by morphine induces PI3K/Akt activation during HIV-1 infection. Morphine had no significant impact on pAkt expression in HIV-infected THP-1/PMA macrophages (**Figure 8A**). In contrast, morphine significantly enhanced pAkt expression in HIV-infected MOR-THP-1/PMA macrophages, which was suppressed by both naloxone and the PI3K inhibitor, wortmannin (**Figure 8B**). Morphine modestly enhanced pAkt expression in iPSC-derived microglia which was reversed by naloxone and wortmannin (**Figure 8C**). We next investigated if inhibition of the PI3K/Akt pathway would abrogate morphine-induced HIV-1 infection enhancement. THP-1/PMA macrophages and iPSC-derived microglia were pretreated with wortmannin in the presence or absence of morphine for 24 hours before initiating HIV-1 infection. Importantly, wortmannin treatment did not significantly impact cell viability in iPSC-derived microglia (**Supplementary Figure S3A**), THP-1/PMA (**Supplementary Figure S3B**), and MOR-THP-1/PMA macrophages (**Supplementary Figure S3C**). Though impact of wortmannin on p24^Gag^ secretion in the absence of morphine was minimal and not significant in all cell types (**Figures 8D-F**), in the presence of morphine, wortmannin treatment significantly reduced p24^Gag^ production in MOR-THP-1/PMA macrophages (**Figure 8E**) and iPSC-derived microglia (**Figure 8F**), suggesting that enhanced p24^Gag^ production by morphine in MOR expressing cells is mediated by the PI3K/Akt pathway.

**Figure 9:**
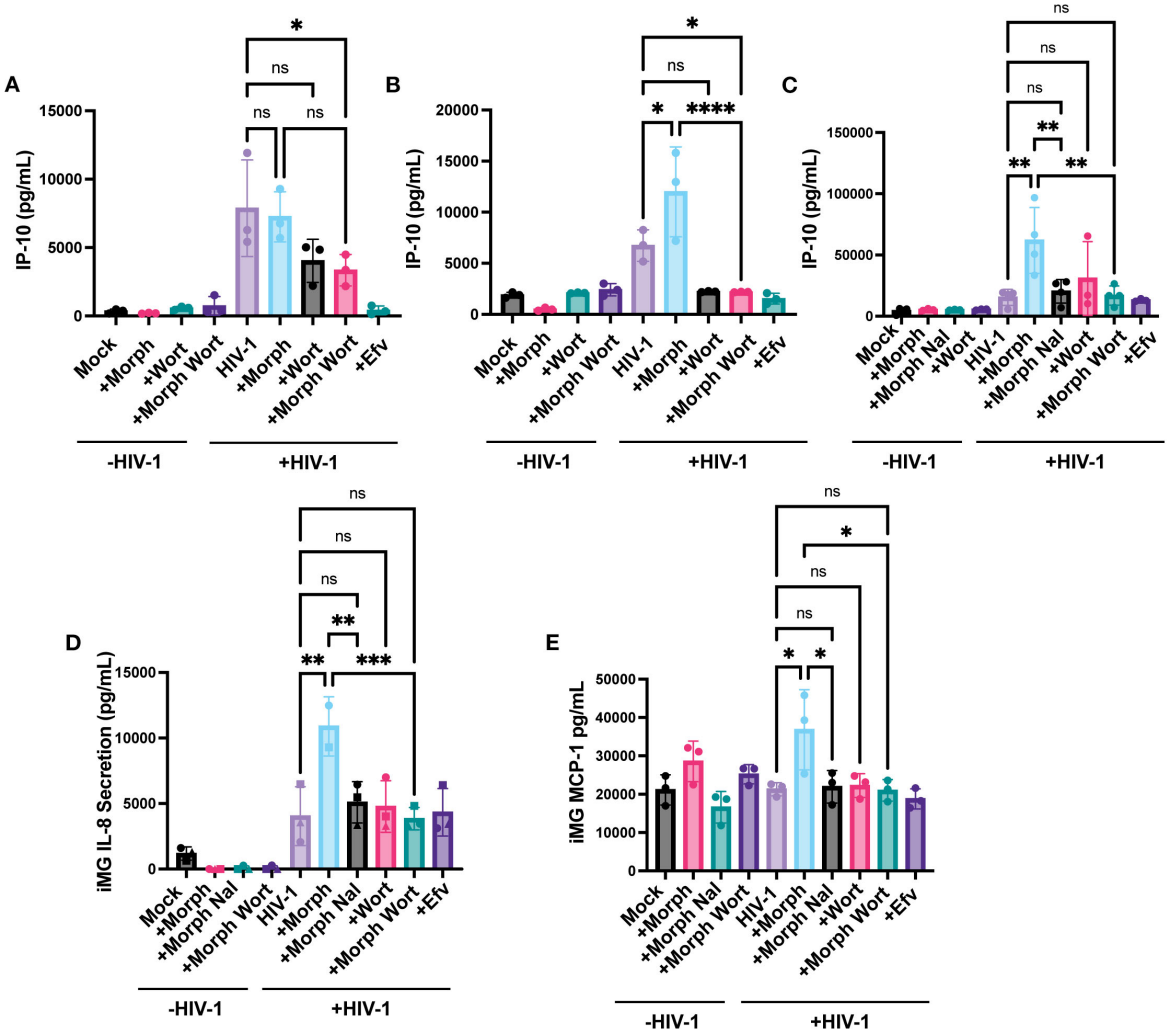
PI3K Inhibition suppresses HIV and morphine-induced inflammatory responses. THP-1/PMA **(A)**, MOR-THP-1/PMA **(B)** macrophages and iPSC-derived microglia **(C-E)**, were pretreated with DMSO (vehicle control) or naloxone (1 µM) or wortmannin (0.1 µM) for 1 hr prior to morphine (1 µM) treatment for 24hr, and infected with either LaiΔEnvGFP/G (MOI 1), in the presence of SIVmac Vpx VLPs (5 ng p27) or Lai-YU2-env (MOI 1), respectively. Supernatants were harvested 3 days post-infection for quantification of IP-10 secretion in **(A)** THP-1/PMA, **(B)** MOR-THP-1/PMA macrophages, and **(C)** iPSC-derived microglia, or **(D)** IL-8 secretion and **(E)** MCP-1 secretion in iPSC-derived microglia by ELISA. The means +/- SEM are shown, and each symbol represents an independent experiment. p values: one-way ANOVA followed by the Tukey-Kramer posttest; *, p< 0.05; **, p< 0.01; ***, p< 0.001; ****, p< 0.0001 **(A–E)**.

Finally, we investigated whether PI3K/Akt inhibition affects morphine and virus-induced inflammatory responses. Wortmannin pre-treatment significantly inhibited morphine-mediated enhancement of IP-10 secretion by ~7.7-fold in HIV-infected MOR-THP-1/PMA macrophages (**Figure 9B**) and ~2.8-fold in iPSC-derived microglia (**Figure 9C**) but had a negligible impact on IP-10 secretion in HIV-1-infected THP-1/PMA macrophages (**Figure 9A**). Additionally, wortmannin pre-treatment attenuated IL-8 and MCP-1 secretion (**Figures 9D, E**) in morphine-treated HIV-1-infected iPSC-derived microglia. Collectively, these findings suggest that opioids promote the induction of inflammatory responses in HIV-infected microglia via MOR activation and that PI3K inhibitors can suppress the synergistic enhancement of inflammatory responses by morphine-induced activation of MOR signaling pathways and HIV-1 infection.

## Discussion

In this study, we utilized multiple cell models to demonstrate that morphine enhances HIV-1 infection and promotes HIV-1 infection-induced inflammatory responses in MOR-expressing cells. We report cell surface expression of MOR in iPSC-derived microglia, similar to that observed in CNS-resident microglia (35, 85), which correlated with the ability of morphine to enhance HIV-1 infection in MOR+ iPSC-derived microglia but not in MOR-deficient macrophages. Exogenous expression of MOR in THP-1/PMA macrophages promoted morphine-mediated enhancement of HIV-1 infection. Importantly, morphine-dependent enhancement of HIV-1 infection was attenuated upon pre-treatment with a MOR antagonist, naloxone. Taken together, these data suggest that morphine may trigger MOR-dependent signaling in modulating HIV-1 infection in microglia. Previous studies have shown that morphine can modulate CXCR4 and CCR5 expression in myeloid cells (86, 87) and lymphocytes (88). Similarly, endogenous ligands such as endomorphins and endorphins that activate MOR may have similar effects of enhancing HIV-1 infection in microglia *in vivo* (89, 90). While morphine may enhance CCR5 expression in iPSC-derived microglia and promote HIV-1 entry, proviral effects of morphine were also observed at post-entry steps of revere transcription and integration since morphine also induced VSV G-pseudotyped HIV-1 infection in MOR THP-1/PMA macrophages (**Figures 2**, **3**). As opposed to MOR-THP1/PMA macrophages, there was a trend but not statistically significant enhancement of early RT in iPSC-microglia by morphine (**Figure 3A**), suggesting that morphine might also modulate post RT steps to enhance HIV-1 replication in iPSC-microglia. In contrast, morphine significantly enhanced reverse transcription in MOR THP-1/PMA macrophages (5-fold increase in late RT products, **Figure 3**), thus contributing to the observed increases in the numbers of intact proviruses (**Figure 4**) in these cells. Previous studies have reported conflicting findings on the immuno-modulatory effects of opioids and their ability to impact HIV-1 infection in monocytes and macrophages (87, 91, 92). Though MOR mRNA has been detected at low levels in human MDMs (38, 93), none of the studies have definitively shown functional cell surface MOR expression in macrophages (37, 38, 94–96). Further, our findings show that MDMs do not express MOR at detectable levels (**Figure 1**). It remains possible that the previously reported immune-modulatory effects of morphine in macrophages might be attributed to TLR4 or non-classical opioid receptor signaling upon morphine exposure (97).

Clinically, people who have frequent ART interruptions have higher HIV-1 proviral DNA reservoirs than people with fewer ART interruptions (98). People with HIV-1 who inject drugs have higher incidences of ART interruption (99) and might have a higher risk of elevated HIV proviral DNA levels. Studies in PWH who inject heroin, which is metabolized into morphine, reported minor increases in intact HIV proviral DNA in PBMCs (98). Additionally, another study has shown that morphine increases the size of SIV reservoirs in brain resident CD11b+ macrophages in rhesus macaques (100), though the effects of opioids on viral reservoirs in the CNS have not been well-characterized in PWH. Previous studies have suggested that opioids can suppress type I IFN responses and expression of antiviral restriction factors (38), such as SAMHD1 and APOBEC3. Suppression of these anti-viral responses might enhance the efficiency of HIV-1 reverse transcription and provirus establishment in MOR-expressing microglia. It should be noted that the ratio of defective to intact HIV-1 proviruses was considerably lower in iPSC-derived microglia, compared to that observed in human viremic brain tissues (>90% of HIV-1 genomes are defective *in vivo*). *In vivo* findings likely reflect chronic HIV-1 infection for several months to years in the presence of ART, whereas our data reflect only acute infection conditions in the absence of ART.

### Morphine alters HIV-1 transcriptomic landscape in a MOR dependent manner

Morphine exposure led to the increased expression of LTR-containing HIV-1 transcripts, though interestingly, enhancement of unspliced icRNA expression was greater than that observed with multiply-spliced transcripts in microglia and MOR-THP1/PMA macrophages. HIV-1 LTR contains numerous binding sites for transcription factors such as NF-kB, NFAT, AP-1, and CREB (101). Previous studies have suggested that opioids enhance gene expression by activating transcription factors such as NF-κB (102–105) and CREB (103), which may enhance HIV-1 transcription rates in infected microglia, though such a mechanism would not account for the selective enhancement of HIV-1 icRNA expression in morphine-treated microglia. Additionally, since morphine enhanced the ratio of unspliced full-length icRNA and 5’LTR+Nef+Gag transcripts compared to all other LTR-containing transcripts in microglia, morphine might have an additional impact on post-transcriptional mechanisms that control HIV-1 transcript diversity.

Complex alternative splicing of HIV-1 RNA in the nucleus results in the over 40 differentially spliced viral transcripts, some of which encode for the viral accessory proteins and env (106). Spliced RNAs are exported through the NXF1-dependent nuclear export pathway (107). To facilitate nuclear export of unspliced full-length viral RNA, which is utilized for translation of Gag and Gag-Pol polyproteins or used as viral genomic RNA packaged into assembling virions, HIV-1 employs the Rev-CRM1 pathway to suppress RNA splicing and promote nuclear export (106). Morphine may potentially impact either one or multiple of these post-transcriptional RNA processing steps to skew the viral transcript population towards expression of immunostimulatory unspliced icRNA transcripts. For instance, morphine has been shown to modulate the abundance of m6A epitranscriptomic modifications (108, 109) by downregulating the expression of RNA demethylases or m6A erasers, FTO and Alkbh5 (109). Some studies have shown that m6A methylation at 3’ splice sites of various cellular genes inhibits RNA splicing (110). RNA methylation at the major splice donor site in HIV-1 5’UTR has been suggested to contribute to reduced splicing efficiency and increased nuclear abundance of HIV-1 unspliced RNA (111–114). Thus, the ability of morphine to modulate both transcriptional and post-transcriptional regulatory mechanisms might contribute to the selective enhancement of full-length unspliced HIV-1 icRNA expression in microglia.

### Morphine treatment enhances innate immune responses during HIV-1 infection

Previous studies by us and others have demonstrated that sensing of HIV-1 icRNA by MDA5 triggers MAVS-dependent innate immune responses and inflammatory cytokine secretion in macrophages and microglia (80, 115–117). Despite ART, HIV-1 RNA has been detected in CNS and CSF of PWH (118–122). Innate immune sensing of persistent HIV-1 icRNA expression and morphine-induced MOR signaling in microglia might contribute to the synergistic increases in inflammatory responses. Our results (**Figure 7**) and previously published studies (123, 124) suggest that opioid signaling through MOR activates the PI3K/Akt pathway. Interestingly, PI3K inhibitor wortmannin suppressed morphine-induced enhancement of p24^Gag^ secretion (**Figure 8**) and IP-10 expression (**Figure 9**) in HIV-infected microglia and MOR-THP-1/PMA macrophages. We hypothesize that the putative synergy between morphine – MOR signaling and HIV icRNA-induced proinflammatory responses might define the molecular basis for HIV and opiate co-exposure-induced neuroinflammation and neurotoxicity. Importantly, opioid antagonists or PI3K/Akt pathway inhibitors might be novel therapeutic modalities to suppress chronic neuroinflammation in PWH using injection drugs (22, 125–128).

### Limitations of the study

The experimental model under study in this report addresses consequences of morphine pre-treatment on HIV-1 infection establishment and effects in HIV-1 infection-induced innate immune responses, and most likely represents the scenario of individuals with opioid use disorder who later acquire HIV. This study does not address the consequences of morphine exposure post-infection establishment that might be reflective of PWH who use opioids. Such experimental conditions can be investigated in the future by adding morphine during and after infection establishment in microglia. Finally, there are some discrepancies between the cell models where morphine significantly enhances reverse transcription in THP-1/PMA macrophages, but only trends to enhance reverse transcription in iPSC-derived microglia in a MOR-dependent manner. This discrepancy reflects that morphine may have other reverse transcription-independent effects on HIV-1 infection enhancement in iPSC-derived microglia that differ from the MOR THP-1 cell line.

## Conclusions

This study demonstrated for the first time that morphine treatment in human microglia enhances HIV-1 infection. Morphine enhancement of HIV-1 infection is specific to CNS-derived microglia and MOR-expressing cells. Morphine had an additional selective impact on enhanced HIV-1 icRNA expression, sensing of which leads to the induction of inflammatory responses. These findings highlight how opioid use contributes to elevated neuroinflammation and risk for neurodegenerative disorders in people with HIV-1. Therapeutics targeting the PI3K/Akt pathway may reduce neurocognitive disorders in PWH.

## Data Availability

The original contributions presented in the study are included in the article/**Supplementary Material**. Further inquiries can be directed to the corresponding author.
