# Supplementary figures and images for: Mu opioid receptor activation in microglia enhances HIV-1 infection and HIV-infection-induced inflammatory responses
Source: Front Immunol. 2025 Oct 6;16:1628872. doi: 10.3389/fimmu.2025.1628872 (PMC12536016; doi:10.3389/fimmu.2025.1628872)

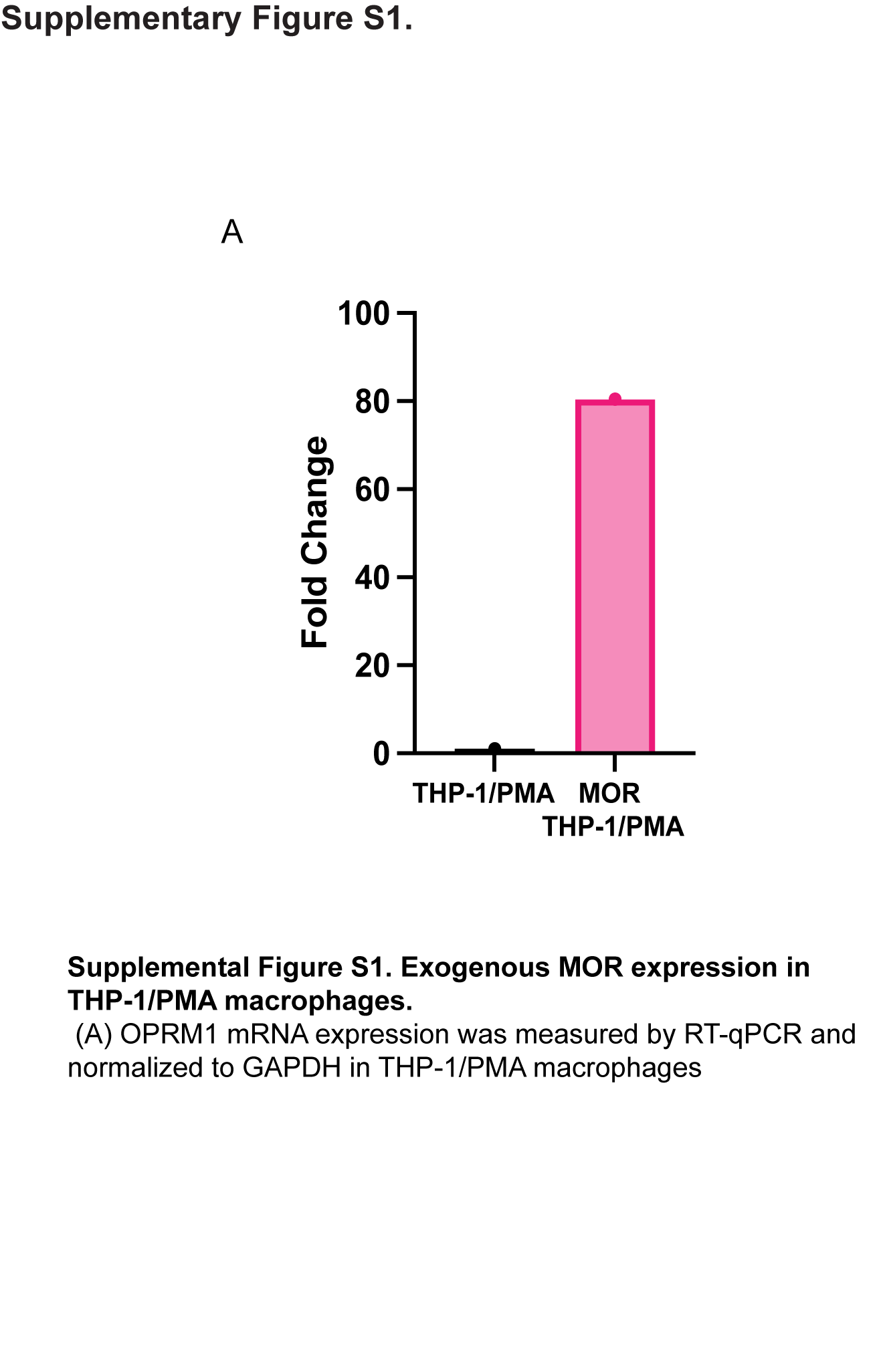

Supplement: Supplementary file 1 [file Image1.tif]

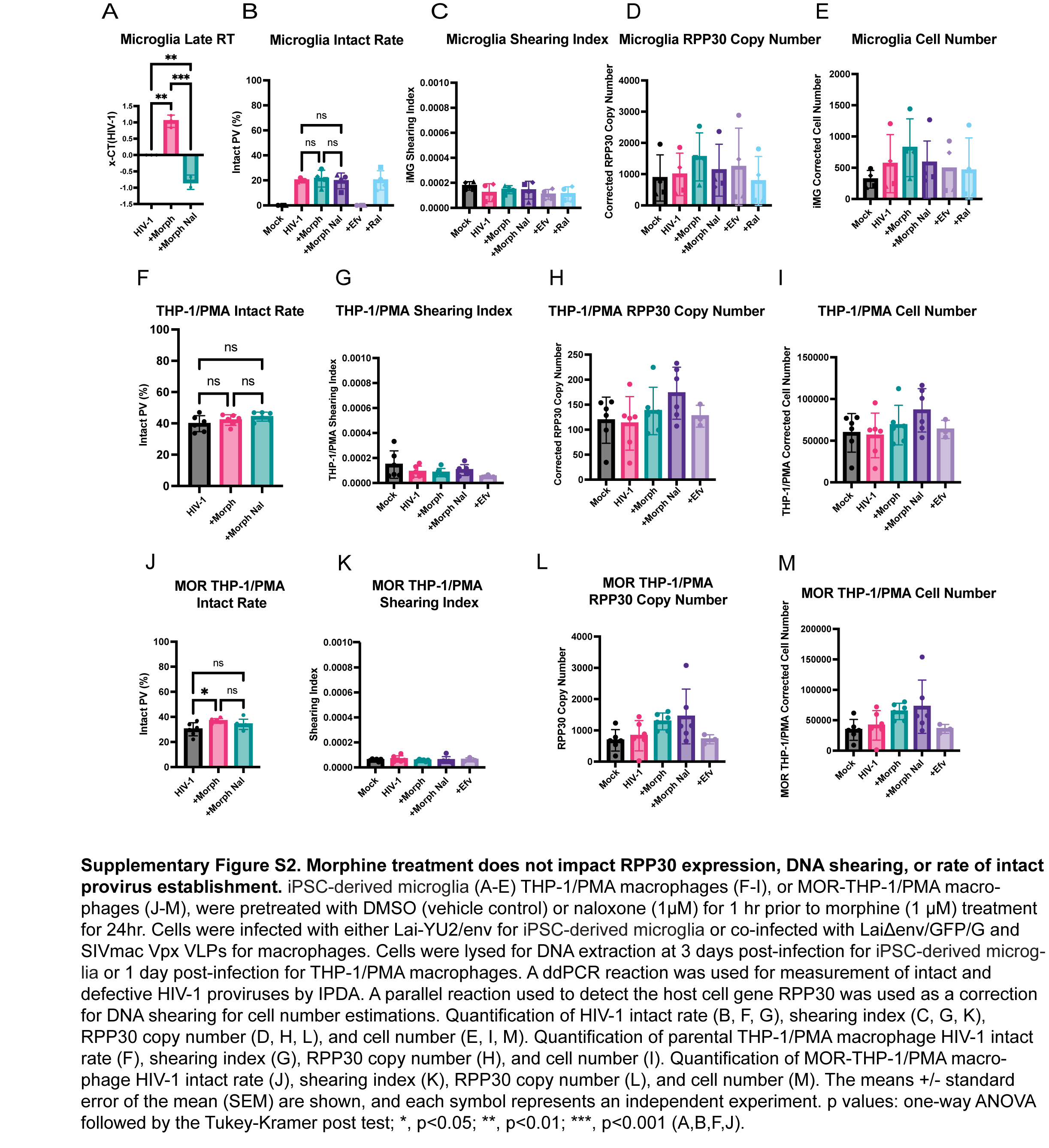

Supplement: Supplementary file 2 [file Image2.tif]

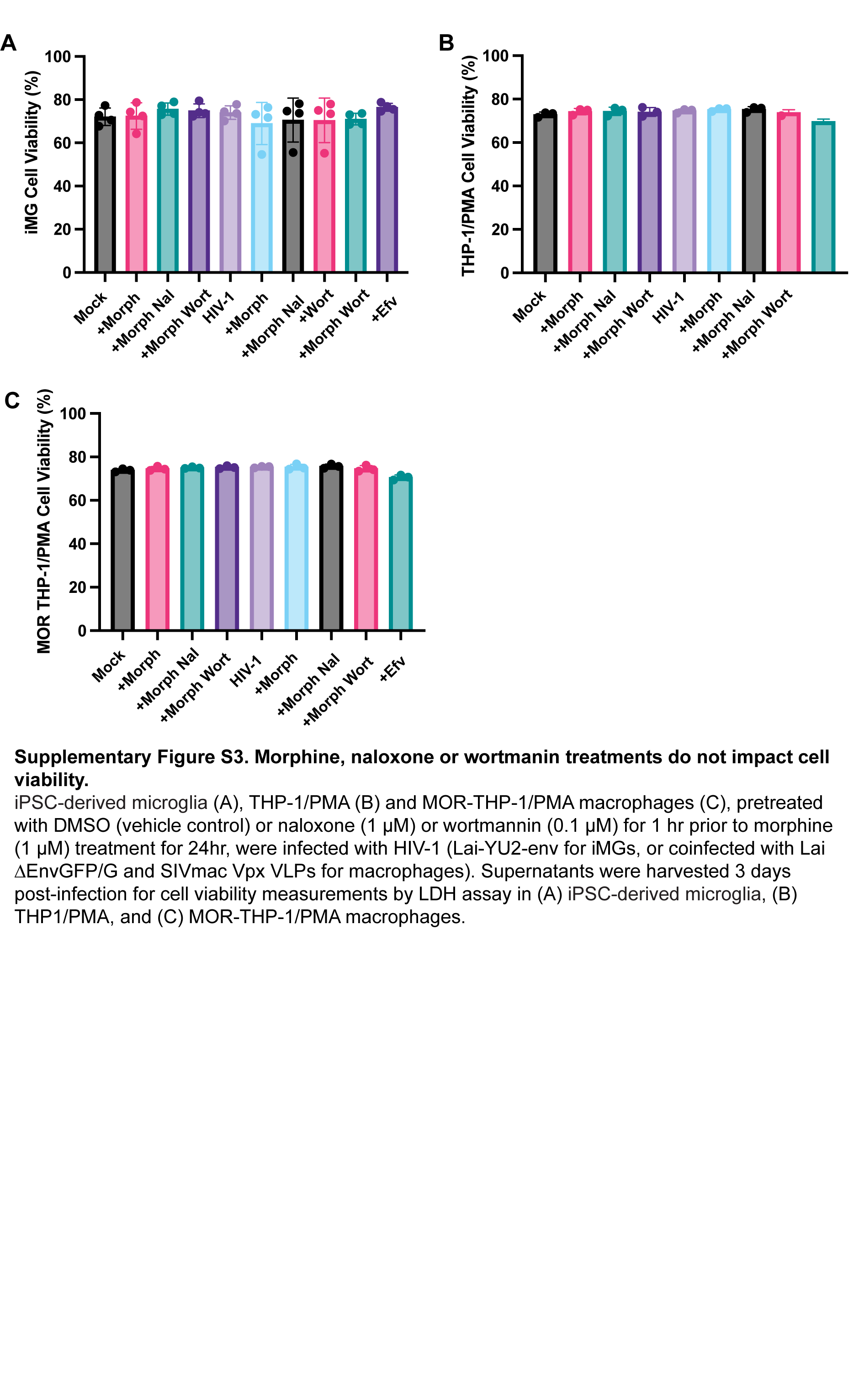

Supplement: Supplementary file 3 [file Image3.tif]
